# C-Reactive Protein as a Predictive Marker for Anastomotic Leak Following Restorative Colorectal Surgery in an Enhanced Recovery After Surgery Program

**DOI:** 10.1007/s11605-023-05798-3

**Published:** 2023-08-09

**Authors:** Joseph Do Woong Choi, Charlotte Kwik, Aswin Shanmugalingam, Lachlan Allan, Toufic El Khoury, Nimalan Pathmanathan, James Wei Tatt Toh

**Affiliations:** 1 Department of Colorectal Surgery, Westmead Hospital. Corner Hawkesbury Road and Darcy Roads, Westmead, NSW Australia; 2 Faculty of Medicine and Health, The University of Sydney, Sydney, NSW Australia; 3 School of Medicine, University of Notre Dame, Sydney, NSW Australia

**Keywords:** Anastomotic leak, C-reactive protein, Colorectal surgery, Cutoff level

## Introduction

Anastomotic leak (AL) remains a serious complication following restorative colorectal surgery. There are published studies on the use of c reactive protein (CRP), procalcitonin, and white cell count (WCC) to predict AL.^1^ However, most are based on heterogeneous retrospectively collected data.

The aims were to assess the role of CRP in the early detection of AL after elective colorectal surgery in the setting of an enhanced recovery after surgery (ERAS) program, and to determine the most predictive postoperative day (POD) cutoff CRP value.

## Methods

Data were derived from a single institution, prospectively collected database. Three hundred sixty-one patients underwent elective colorectal surgery with primary anastomosis (with or without defunctioning stoma) from January 2017 to December 2022. Emergency and palliative procedures were excluded.

Serum CRP was measured daily between POD 1 and 5. AL was defined radiologically and/or intraoperatively as “a defect of the intestinal wall at the anastomotic site, leading to a communication between the intra and the extra-luminal compartments”.^2^

Data are presented as median, inter-quartile ranges, and percentages. Categorical data were analysed using the Chi Square test. Quantitative variables were analysed using the Mann–Whitney *U* test. Receiver operating characteristic (ROC) curve was used to determine cutoff values, sensitivity, specificity, positive predictive values (PPV), and negative predictive values (NPV). *P* values < 0.05 were considered significant.

## Results

**Fig. 1:**
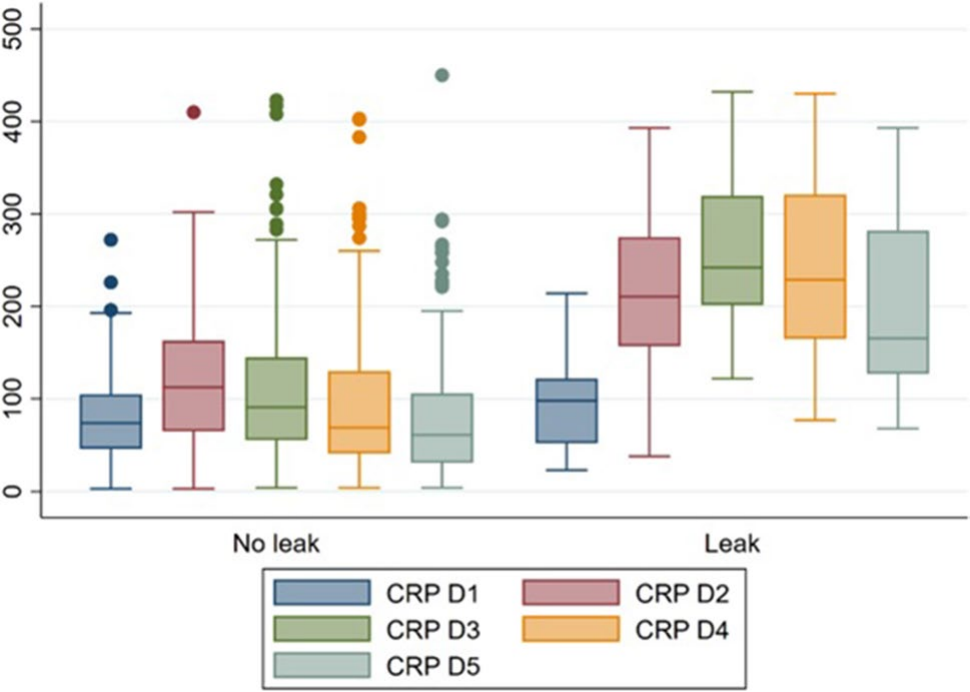
Box and whisker plot of POD 1 to 5 CRPs comparing no AL versus AL patients

**Fig. 2:**
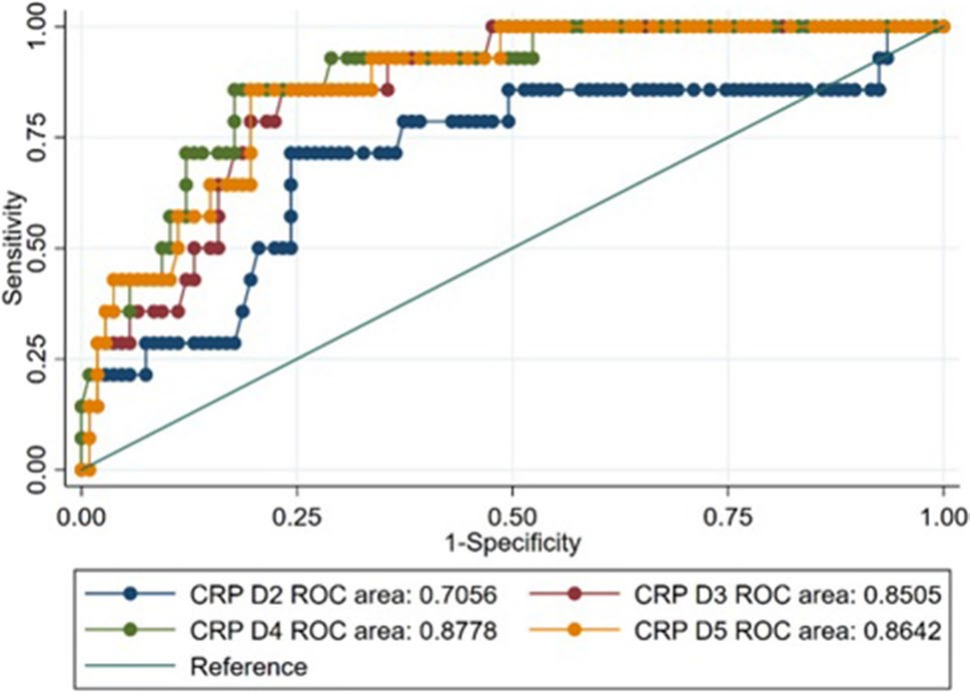
ROC curves for AL patients at POD 2 to 5

The incidence of AL was 4.4% (16/361), with an overall mortality of 1.4% (5/361), 6.3% (1/16) in the AL group, and 1.2% (4/345) in the no AL group. The median CRP for POD 1, 2, 3, 4, and 5 in the AL group was 96, 211, 242, 229, and 166 mg/L, respectively (normal range ≤ 4 mg/L). Statistical significance was observed at POD 2–5 when compared to the no AL group (Table 1 and Fig. 1). The box and whisker plot highlights that CRP values at POD3 were the best predictor for AL (Fig. 1). After plotting ROC curves, CRP at POD3 was the most accurate in predicting anastomotic leak, with cutoff levels < 182 mg/L on POD3 a good predictor of no AL (sensitivity 88%, specificity 87%, PPV 28.6%, NPV 99.1%) (Fig. 2).

**Table 1:** Patient and clinical characteristics

Characteristic	Total (*n* = 361)	No AL	AL	*P* value
Gender, *n* (%)				0.837
Male	167 (46.3%)	160 (46.4%)	7 (43.8%)	
Female	194 (53.7%)	185 (53.6%)	9 (56.2%)	
BMI (median, IQR)	27.7 (23.95–31.85)	27.8 (24–31.95)	27 (23.45–29.4)	0.5430
HbA1c (median, IQR)	5.5 (5.2–6)	5.5 (5.2–6)	6.1 (5.35–7.25)	0.0784
Hospital stay (median, IQR)	6 (4–9)	6 (4–8)	20 (11.5–46.5)	< 0.00001
Procedure, *n* (%)				0.021
Right hemicolectomy	111 (31.0%)	111 (32.5%)	0 (0%)	
Transverse colectomy	4 (1.1%)	4 (1.2%)	0 (0%)	
Left hemicolectomy	16 (4.5%)	15 (4.4%)	1 (6.25%)	
Anterior resection (height not specified)	10 (2.8%)	10 (2.9%)	0 (0%)	
High anterior resection	95 (26.3%)	91 (26.7%)	4 (25%)	
Low anterior resection	47 (13%)	42 (12.3%)	5 (31.25%)	
Ultralow anterior resection	42 (11.6%)	37 (10.8%)	5 (31.25%)	
Total proctocolectomy and pouch with defunctioning ileostomy	16 (4.5%)	16 (4.7%)	0 (0%)	
Other restorative procedures	17 (4.8%)	16 (4.7%)	1 (6.25%)	
Diverting stoma				0.735
Diverting ileostomy	79	76 (22.35%)	3 (18.75%)	
No diverting ileostomy	277	264 (77.65%)	13 (81.25%)	
Approach				0.557
Open	35 (9.8%)	34 (10%)	1 (6.3%)	
Laparoscopic	239 (67.1%)	229 (67.4%)	10 (62.5%)	
Converted to open	32 (9%)	29 (8.5%)	3 (18.8%)	
Hand assisted laparoscopic (hybrid)	50 (14%)	48 (14.1%)	2 (12.5%)	
Indication: Colorectal cancer (CRC) vs non-CRC				1.000
CRC	285 (79%)	272 (78.8%)	13 (81.3%)	
Non-CRC Crohn’s disease Ulcerative colitis Diverticulitis Volvulus Others*	76 (21%) 6 (1.7%) 3 (0.8%) 33 (9.1%) 1 (0.3%) 32 (8.9%)	73 (21.2%) 6 (1.7%) 3 (0.9%) 32 (9.3%) 1 (0.3%) 30 (8.7%)	3 (18.7%) 0 (0%) 0 (0%) 1 (6.2%) 0 2 (12.5%)	
Superficial surgical site infection	21 (5.8%)	18 (5.2%)	3 (18.75%)	0.024
No superficial surgical site infection	340 (94.2%)	327 (94.8%)	13 (81.25%)	
Deep surgical site infection	8 (2.2%)	5 (1.4%)	3 (18.8%)	< 0.0001
No deep surgical site infection	353 (97.8%)	340 (98.6%)	13 (81.2%)	
Organ/space occupying infection	7 (1.9%)	3 (0.8%)	4 (25%)	< 0.0001
No organ/space occupying infection	354 (98.1%)	342 (99.2%)	12 (75%)	
Urinary tract infection	20 (5.5%)	18 (5.2%)	2 (12.5%)	0.213
No urinary tract infection	341 (94.5%)	327 (94.8%)	14 (87.5%)	
Pneumonia	9 (2.5%)	7 (2%)	2 (14.3%)	0.009
No pneumonia	352 (97.5%)	338 (98%)	14 (87.5%)	
Deep vein thrombosis	5 (1.4%)	2 (0.6%)	3 (18.8%)	< 0.0001
No deep vein thrombosis	356 (98.6%)	343 (99.4%)	13 (81.2%)	
Prolonged ileus > POD3	45 (12.5%)	40 (11.6%)	5 (31.3%)	0.02
No prolonged ileus	316 (87.5%)	305 (88.4%)	11 (68.7%)	
CRP POD1 (median, IQR)	75 (46–107)	75 (46–107)	96 (52–113)	0.5836
CRP POD2 (median, IQR)	116 (65–170)	114 (65–165)	211 (151–249)	0.0015
CRP POD3 (median, IQR)	96 (57–153)	91 (56–145)	242 (210–308)	< 0.00001
CRP POD4 (median, IQR)	80 (43–141)	69 (41–131)	229 (179–302)	< 0.00001
CRP POD5 (median, IQR)	68 (35–127)	62 (31–110)	166 (129–273)	< 0.00001

*AL*, anastomotic leak; *BMI*, body mass index; *IQR*, interquartile range; *endometriosis, other non-colorectal malignancies including gynaecological malignancies, etc.

## Discussion

The study showed that serum CRP levels started to downtrend from POD 3 in patients who did not have AL. However, in the AL group, the median value of CRP on POD 3 was 242 mg/L, and this remained high on POD 4–5. This trend was also seen in other studies reporting AL, with peak CRP ranging between 102 and 254.7 mg/L at POD 3.^3,4^

The study also highlights that CRP levels at POD 3 was a good predictor of no AL. The advantage of POD 3 CRP testing is the early detection of AL in patients who do not have clinical manifestations of AL. In patients with a high CRP on POD3, a higher index of suspicion for AL based on high CRP values may trigger imaging if patient develops any signs or symptoms. Su’a et al. analysed 11 studies on AL and identified a wide variation in CRP cutoff values, ranging from 94 to 190 mg/L for POD 3–4.^5^ A recent prospective study involving 113 patients demonstrated that the cutoff CRP value of 166 mg/L at POD 3 had the greatest area under the ROC curve (AUC) of 0.853, with an 81.81% sensitivity, 82.42% specificity, and NPV of 93.8%.^6^

The limitations of this study include small cohort and unblinded study where investigators used CRP, other biomarkers, and clinical assessment to guide postoperative management. This may have resulted to a bias towards the utility of CRP to detect AL.

## Conclusion

Patients with a CRP cutoff value of < 182 mg/L at POD 3 may be earmarked for early discharge if clinically appropriate.
